# Cost-effectiveness of new pneumococcal conjugate vaccines in Turkey: a decision analytical model

**DOI:** 10.1186/1472-6963-12-386

**Published:** 2012-11-09

**Authors:** Mustafa Bakır, Özden Türel, Oleksandr Topachevskyi

**Affiliations:** 1 Department of Pediatrics and Pediatric Infectious Diseases, Marmara University School of Medicine Hospital, Altunizade, Pendik, Istanbul, 34662, Turkey; 2 Department of Pediatrics and Pediatric Infectious Diseases, Bakirkoy Maternity and Children’s Research Hospital, Istanbul, Turkey; 3 GlaxoSmithKline Vaccines, Wavre, Belgium

## Abstract

**Background:**

*Streptococcus pneumoniae* infections, which place a considerable burden on healthcare resources, can be reduced in a cost-effective manner using a 7-valent pneumococcal conjugate vaccine (PCV-7). We compare the cost effectiveness of a 13-valent PCV (PCV-13) and a 10-valent pneumococcal non-typeable *Haemophilus influenzae* protein D conjugate vaccine (PHiD-CV) with that of PCV-7 in Turkey.

**Methods:**

A cost-utility analysis was conducted and a decision analytical model was used to estimate the proportion of the Turkish population <10 years old that would experience 10 mutually exclusive outcomes over the course of 1 year from a perspective of a healthcare system. Model outcomes were adjusted according to the population demographics and region-specific serotype distribution in Turkey. Health outcomes and direct healthcare costs were simulated for PCV-7, PCV-13 and PHiD-CV.

**Results:**

PCV-13 and PHiD-CV are projected to have a substantial impact on pneumococcal disease in Turkey versus PCV-7, with 2,223 and 3,156 quality-adjusted life years (QALYs) and 2,146 and 2,081 life years, respectively, being saved under a 3+1 schedule. Projections of direct medical costs showed that a PHiD-CV vaccination programme would provide the greatest cost savings, offering additional savings of US$11,718,813 versus PCV-7 and US$8,235,010 versus PCV-13. Probabilistic sensitivity analysis showed that PHiD-CV dominated PCV-13 in terms of QALYs gained and cost savings in 58.3% of simulations.

**Conclusion:**

Under the modeled conditions, PHiD-CV would provide the most cost-effective intervention for reducing pneumococcal disease in Turkish children.

## Background

Infection with *Streptococcus pneumoniae* can result in invasive pneumococcal disease (IPD) (e.g. meningitis and bacteremia) and non-invasive pneumococcal disease (e.g. community-acquired pneumonia [CAP] and acute otitis media [AOM]). In Turkey in 2000, lower respiratory infections were the fifth most common cause of death in the total population (accounting for 4% of deaths), and the second most common cause of death among 0-14-year olds (14% of deaths); and meningitis was the fifth most common cause of death among 0-14-year olds (3% of deaths) [1]. Results in terms of disability-adjusted life years (DALYs) were similar [1], showing that these infections are a serious cause of morbidity as well as mortality.

Based on the high burden of pneumococcal diseases (particularly in young children), increasing antibiotic resistance, and the efficacy [2,3], safety [2] and cost-effectiveness [4] of a 7-valent pneumococcal conjugate vaccine (PCV-7; Pfizer), the World Health Organization (WHO) recommended in 2007 that pneumococcal vaccination should be included in national childhood immunization programs [5]. This was implemented in Turkey in November 2008 [6]. Other vaccines recently licensed in Turkey are a 13-valent pneumococcal conjugate vaccine (PCV-13; Pfizer) and one that contains 10 pneumococcal serotypes and a carrier protein derived from non-typeable *Haemophilus influenzae* (NTHi): pneumococcal non-typeable *H. influenzae* protein D conjugate vaccine (PHiD-CV; GSK Vaccines). The latter has the added advantage of providing protection against AOM caused by NTHi [7], which causes around a third of AOM cases (with another third being due to *S. pneumoniae*[8]).

In 2003, the World Bank issued a report highlighting the inadequacies of health services in Turkey, and the poor health status of Turkish people compared to those in other middle-income countries [9]. The main aims outlined in the report were to improve access to health services, quality of care and health outcomes; and increase cost-effectiveness [9]. Since adopting the Health Transformation Program, resource use in Turkey has been optimized and the health system has become more effective, efficient and equitable [6]. In 2009, the Turkish budget for vaccination was 205 million Turkish Lira (TL) [6]. By comparison, a budget of only 14 million TL was allocated for vaccination in 2002 [6]. Further improvement of Turkey’s vaccination program was one of the priorities set out by a Biennial Collaborative Agreement between the Ministry of Health of Republic of Turkey and the Regional Office for Europe of the WHO in 2010 [10]. The aims were to maintain polio-free status, eliminate measles and rubella, provide equitable access to vaccines, and include new immunization products and technologies for vaccine-preventable diseases [10].

Immunization is generally considered to be one of the most cost-effective health investments [11]. In Turkey, children are routinely vaccinated against tuberculosis, hepatitis B, diphtheria, pertussis, tetanus, *H. influenzae* type b, polio, measles, mumps, rubella, and meningitis, as well as receiving PCV-7 [6,12]. However, with the introduction of the newer pneumococcal vaccines, the relative cost-effectiveness of PCV-7, PCV-13 and PHiD-CV needs to be ascertained. Therefore, the objective of this paper is to estimate the public health and economic impact of changing from PCV-7 to either PCV-13 or PHiD-CV in Turkey.

## Methods

### Model overview

**Figure 1:**
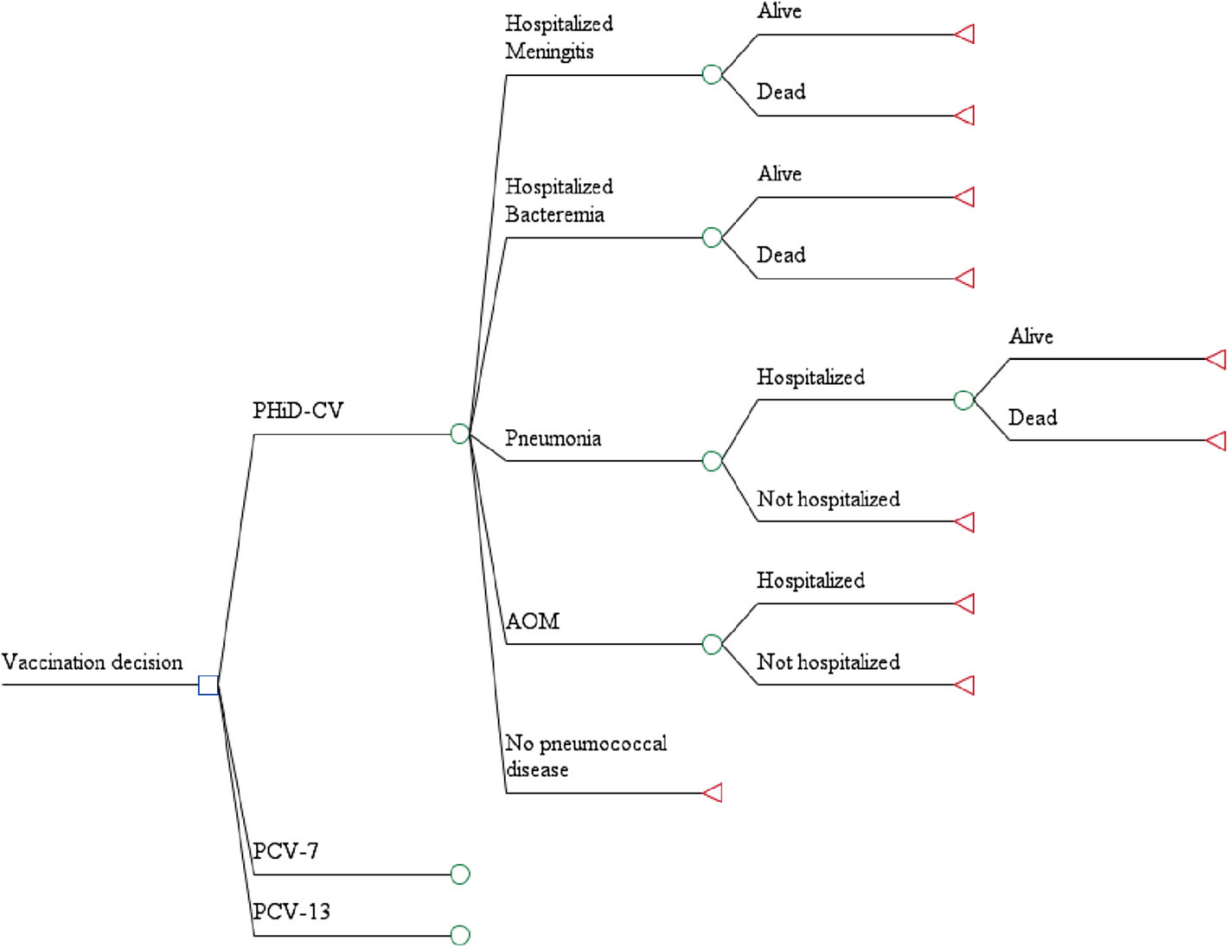
**Model structure: decision tree framework.** The 10 mutually exclusive health outcomes for pneumococcal-related disease are represented by the 10 branches of the decision analytical model. Although the model is capable of estimating the disutility of long-term sequelae, these were not included in the presented analysis, due to a lack of accurate, region-specific data. AOM = acute otitis media, PCV-7 = 7-valent pneumococcal conjugate vaccine, PCV-13 = 13-valent pneumococcal conjugate vaccine, PHiD-CV = pneumococcal non-typeable *Haemophilus influenzae* Protein D conjugate vaccine.

The health economic model was used to conduct a cost-utility analysis from the perspective of a healthcare system. This model was derived from a population-based model previously described by De Wals et al. [13]. The model is comprised of a decision tree framework that terminates in 10 mutually exclusive pneumococcal-related health outcomes (Figure 1). For each vaccination program considered, the proportion of the Turkish population arriving at each health outcome over the course of a 1-year period is estimated. Vaccination schedules are assumed to exert a “steady state” effect on the entire population. This implies that the given vaccination program has been established long enough to have a consistent effect year after year. This evaluation estimates the direct impact of vaccination on children at risk aged 0–9 years only. Hence, we assumed that a steady state will have been reached 10 years after vaccination of the first birth cohort. In contrast to traditional lifetime Markov models, our model evaluates cost effectiveness in a single year (once equilibrium has been established). Finally, the model is stratified by a series of age compartments, which enables estimates to be adjusted according to population demographics and age-specific parameters.

The model was used to compare three vaccination scenarios: a currently existing PCV-7 vaccination program compared with PHiD-CV or PCV-13; and direct comparison of PHiD-CV versus PCV-13 given in a 3+1 schedule consisting of three doses in the first 6 months of life, with a booster at 12–15 months of age. For the three vaccination scenarios, all children were assumed to receive the full course of doses with an overall coverage rate of 84%. This estimation was based on data from the US National Immunization Survey 5 years after PCV-7 was introduced [14].

### Epidemiology data

The population of Turkey (estimated at 72 million) and age-specific demographic data were obtained from national census data for the year 2000 adjusted to the current population size [15]. This corresponded with a total vaccinated population (defined as children aged 2–13 months) of 1,400,000 individuals. For children aged <5 years of age, disease incidence of acute episodes and rates of hospitalization and ambulatory cases for IPD and CAP were taken from a previous study of admissions to 12 Istanbul hospitals, which cover 65% of the city’s population [16], and a National Burden of Disease and Cost Effectiveness Study [17] (Table 1). This in turn represents 19% of the overall Turkey population of 70 million people. We assumed that 1/3 of children aged <5 years would experience AOM, as has been reported in studies from other European countries [18,19]. Data specific to Turkey were not available for children aged 5–9 years. Therefore, the incidences of acute IPD, CAP and AOM for these children were based on the average incidences in children aged <5 years in Turkey and the ratio of incidences in children aged <5 and 5–9 years in the UK. Data for IPD, CAP and AOM in the UK were taken from the Health Protection Agency [20], Hospital Episode Statistics [21] and Melegaro and Edmunds [19], respectively. Hospitalization rates for AOM and the proportion of hospitalized AOM cases requiring myringotomy were based on Ministry of Health statistics for the year 2004 [17]. Although AOM can result in various complications, these were not included in the current analysis. The model assumes that 35.9% of AOM cases are attributable to *S. pneumoniae* and that 32.3% of AOM cases are attributable to NTHi. These estimates are calculated from Leibovitz [8], which estimates the cause of disease in children from 23 different datasets across multiple countries. The weighted average across all data sets was calculated for *S. pneumoniae* and NTHi.

**Table 1:** Age-specific incidence of pneumococcal-related diseases, hospitalizations and GP consultations in Turkey per 100,000 population

**Age group (years)**	**Incidence (per 100,000 population) **[16]**,**[17]					
	**Pneumococcal meningitis**	**Pneumococcal bacteremia**	**All-cause pneumonia (hospitalized)**	**All-cause pneumonia (GP)**	**AOM (hospitalized)**	**AOM (GP)**
<1	14.6	45	339	1,356	357.1	33,000
1-<2	10	25	132	528	357.1	33,000
2-<3	5	25	21	84	357.1	33,000
3-<4	5	12.5	21	84	357.1	33,000
4-<5	5	12.5	21	84	357.1	33,000
5-<10	1.2	0.5	15.5	62.4	44.0	20,189

*AOM* acute otitis media, *GP* general practitioner.

The previous study of IPD and CAP <5 years of age in 12 Istanbul hospitals [16] was approved by the Research Ethical Committee of Marmara University School of Medicine. Permission for access to patient charts data was provided by both Turkish Ministry of Health and management of each hospital.

### Quality of life

The model used two types of utility values: normative utility values (the mean utility of living for 1 year for the general population) and the disutilities associated with a disease (the reduction in the normative utility in a patient presenting with a certain condition). Due to the lack of local data in Turkey, all utility values were obtained from international sources and previously published cost-effectiveness studies [19,22,23]. Table 2[24] shows the normative utility allocated to each age, and Table 3[22,23] shows the disutilities the model used for each condition, measured as the amount of utility lost in a year. For most patients, the quality of life reduction associated with the acute episode is limited to a short period of time (1–2 weeks for pneumonia, meningitis or bacteremia; and 1–2 days for AOM). The disutility values generated by these acute episodes are therefore small (0.005-0.023) in annual terms.

**Table 2:** Normative utility values for the general population (men and women)

**Age group (years)**	**Utility value**[24]
<16	0.91
16-24	0.91
25-34	0.91
35-44	0.88
45-54	0.85
55-64	0.79
65-74	0.78
≥75	0.73

**Table 3:** Disutility values

**Disease**	**Disutility value**	**Reference or assumption**
Meningitis (inpatient)*	0.023	[22]
Bacteremia (inpatient)*	0.008	[22]
Pneumonia (inpatient)*	0.008	Assumed to be the same as inpatient bacteremia
Pneumonia (outpatient)*	0.006	[22]
AOM (outpatient)*	0.005	[23]
AOM hospitalized myringotomy*	0.005	Assumed to be the same as for AOM outpatient

*Per episode.

*AOM* acute otitis media.

In our analysis, we used only short-term disutility values for otitis media, which do not take into account pain and hearing loss associated with chronic or recurrent middle ear infection. Therefore, our study is conservative with respect to PHiD-CV. Disutility values for IPD, CAP and AOM were varied by ± 50% in a sensitivity analysis.

### Cost parameters

PHiD-CV, PCV-7 and PCV-13 were assumed to have equal cost per dose (US$30) and per administration (US$3.26). Disease-related costs were estimated from a healthcare payer perspective, whereby only direct medical costs incurred for medical treatment were considered. Costs incurred during treatment of a pediatric patient with pneumococcal meningitis (US$1,000) or pneumococcal bacteremia (US$500), were sourced from a previous study in 12 hospitals in Istanbul [16]. Costs incurred for cases of inpatient pneumonia (US$325) and outpatient pneumonia (US$43), and cases of otitis media requiring hospitalization (US$438) or primary care physician visits (US$30), were sourced from a Turkish National Burden of Disease Study [17].

### Vaccine efficacy

**Figure 2:**
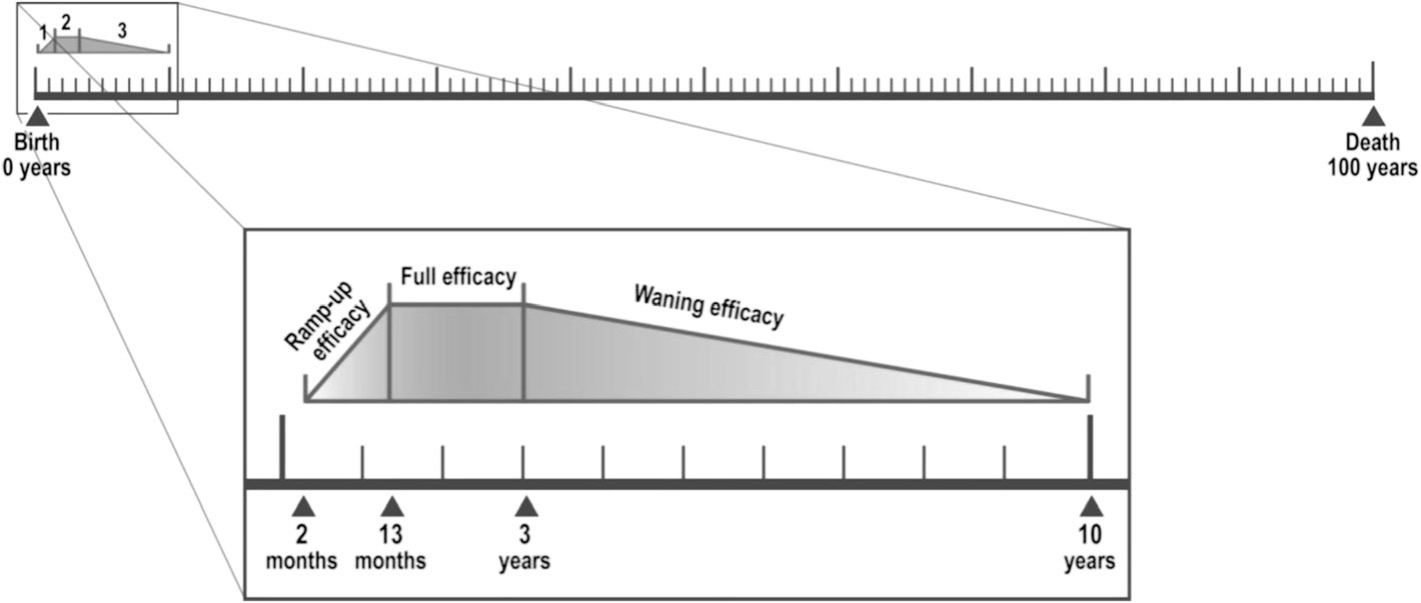
**Modeling age-compartments and vaccine efficacy periods.** Vaccine efficacy was modeled in three phases: initial ramp-up efficacy (2–13 months), full efficacy (13 months-3 years) and waning efficacy (3–10 years).

The model considers individuals to be protected by vaccination from 2 months to 10 years of age. Vaccine efficacy is subdivided into three distinct periods: an initial ramp-up phase that occurs over the course of vaccine administration (2–13 months), a full efficacy phase (13 months-3 years) and a waning efficacy phase (3–10 years) (Figure 2).

Direct vaccine effectiveness was estimated using data collected from prior clinical trials and post-marketing studies performed in the US and Canada, which have been previously summarized for use in health economic analysis [13]. The impact of vaccination on IPD was calculated using serotype-specific vaccine efficacies, adjusted to the age-specific local serotype distribution of *S. pneumoniae* in Turkey [25]. The efficacy of PHiD-CV, PCV-7 and PCV-13 against IPD was assumed to be equal for serotypes shared between the vaccines, and was based on serotype-specific data from a case–control trial of PCV-7 [26]. Vaccine efficacy against IPD for each of the additional serotypes contained in PCV-13 and PHiD-CV was estimated as 94.7%, based on the average reported efficacy of the PCV-7 serotypes [26]. While serotypes 6A and 19A are not covered by PHiD-CV and PCV-7, they are in the same serogroups as 6B and 19F, respectively, which are covered in the vaccines. For PHiD-CV and PCV-7, vaccine effectiveness against IPD caused by serotype 6A was assumed to be 76%, as described in a previous study of PCV-7 in the US [26]. No efficacy against serotype 19A was assumed. Lastly, for PHiD-CV, the impact of vaccination on invasive NTHi disease was also considered. This was achieved by estimating the incidence of invasive NTHi disease. In the absence of Turkish data, it was assumed to be 5% of the incidence of invasive disease, based on data from the Netherlands Reference Laboratory for Bacterial Meningitis [27] and applying the efficacy of the PHiD-CV precursor vaccine against *H. influenza* including NTHi as reported in the POET trial (35.6%) [7].

In the absence of serotype-specific data for pneumonia, the efficacy against ambulatory pneumonia of all three modeled vaccines was assumed to be equal to that observed for PCV-7 in the Northern California Kaiser Permanente study (i.e. 4.3%) [28]. Vaccine efficacy against all-cause (excluding NTHi) pneumonia hospitalizations was estimated as 17.7%, 22.9% and 24.7% for the 7-, 10- and 13-valent vaccines, respectively, as described in a previous health economic analysis of PCVs in the Netherlands [29]. For PHiD-CV, an additional efficacy against hospitalized all-cause pneumonia (1.068%) was also included to account for the vaccine’s potential efficacy against NTHi. This additional efficacy is based on the reported efficacy of the PHiD-CV precursor vaccine against AOM caused by NTHi (35.3%) [7] and an estimation of the incidence of pneumonia hospitalizations due to NTHi (3%) as taken from a review of the literature (i.e. 35.3% × 3% = 1.059%) [30-34].

While NTHi is a recognized cause of pneumonia, the extent of its role remains unclear, since the typing of *H. influenzae* strains is not routine. Within the model, the incidence of NTHi pneumonia was estimated as 3%. The rationale for using a conservative estimation of NTHi pneumonia incidence was informed by the considerable variation amongst estimates reported in the literature [30-34].

Vaccine efficacy against AOM is based on published evidence related to randomized control trials with a 3+1 schedule [7,28,35]. Efficacy is modeled for vaccine type serotypes, non-vaccine type serotypes and *H. influenza*e (including NTHi).

Vaccine efficacy data for PCV-7 are from the per protocol analysis in Eskola et al. [35]. For PHiD-CV, vaccine efficacy data are based on a study of an 11-valent version of PHiD-CV, which was found to reduce AOM episodes caused by *S. pneumoniae* vaccine serotypes by 57.6%, and to reduce episodes caused by NTHi by 35.3% [7]. For PCV-13, the vaccine efficacy against AOM caused by vaccine-type *S. pneumoniae* serotypes was assumed to be equivalent to that of PHiD-CV (57.6%) because the difference in vaccine efficacy (57.2% reported by Eskola et al. [35] for PCV-7) was marginal. We assumed that PCV-7, PHiD-CV and PCV-13 would induce the same level of serotype replacement in non-vaccine serotypes as that observed for PCV-7 (33% increase in non-vaccine serotypes) [35].

Probabilistic sensitivity analyses were performed to assess the effect of uncertainty around the model results. Point estimates used in the base case as input data were replaced by distributions (log normal distribution for vaccine efficacy, beta distribution for disease incidence rates and disutility values and triangular distribution for costs). A total of 1,000 simulations were run, each selecting an input within the distribution. This allows estimation of the range of possible model outcomes.

Univariate sensitivity analyses were performed in order to examine the impact of independently varying the model’s parameters on the costs and quality-adjusted life years (QALYs) gained for PHiD-CV versus PCV-13.

## Results

### Projected health outcomes

The model projects that implementation of a 3+1 vaccination schedule of PHiD-CV or PCV-13 would lead to an estimated reduction in incidence, sequelae and deaths across all diseases compared to PCV-7 in Turkey (Table 4). When the new vaccines are compared directly, PCV-13 was projected to prevent 34 more cases of IPD and 38 more cases of pneumonia hospitalizations than PHiD-CV. However, PHiD-CV was predicted to have a significantly greater impact than PCV-13 on all AOM-related outcomes, including 5,722 hospitalized myringotomy procedures and 192,193 general practitioner (GP) visits. Nevertheless, these results did not translate into a single death difference between PHiD-CV and PCV-13, although a sizeable difference between the number of QALYs saved was apparent, with 933QALYs in favor of PHiD-CV and 65life years (LYs) in favor of PCV-13.

**Table 4:** Projected health outcomes by vaccination strategy for children <10 years old

Health Outcome*	**PCV-7 vaccination**	**PHiD-CV vs PCV-7**	**PCV-13 vs PCV-7**	**PHiD-CV vs PCV-13**
IPD cases	1,991	−190	−225	34
Pneumonia hospitalizations	7,686	−323	−361	38
AOM hospitalizations	24,894	−8,956	−2,234	−5,722
AOM GP visits	2,930,976	−267,212	−75,019	−192,193
Total LYs	1,065,775,026	−2,081	−2,146	65
**Total QALYs**	**906,683,318**	**−3,156**	**−2,223**	**−933**

*Negative numbers represents cases/QALYs/LYs in favor of first comparator.

*AOM* acute otitis media, *GP* general practitioner, *IPD* invasive pneumococcal disease, *LY* life year, *PCV-7* 7-valent pneumococcal conjugate vaccine, *PCV-13* 13-valent pneumococcal conjugate vaccine, *PHiD-CV* pneumococcal non-typeable *Haemophilus influenzae* Protein D conjugate vaccine, *QALY* quality-adjusted life year.

### Projected direct healthcare costs

The projected costs of vaccines and vaccine administration, as well as medical treatment for pneumococcal meningitis, pneumococcal bacteremia, pneumonia and AOM for children aged <10 years are presented in Table 5. The total direct cost of medical treatment, including vaccination costs, under the PCV-7 strategy is projected to be US$260,444,808. Under price parity conditions, PHiD-CV and PCV-13 were projected to reduce this expenditure by US$11.7 million and US$3.4 million, respectively.

**Table 5:** Projected direct costs by vaccination strategy for children <10 years old

	**Costs with PCV-7 vaccination, US$**	Cost savings, US$*		
		**PHiD-CV vs PCV-7**	**PCV-13 vs PCV-7**	**PHiD-CV vs PCV-13**
Vaccine and vaccine administration cost	156,455,040	0	0	0
IPD cost	1,217,684	−112,966	−137,691	24,726
Pneumonia hospitalizations cost	3,939,050	−104,850	−117,264	12,413
AOM cost	98,833,034	−12,500,997	−3,228,848	−8,272,149
**Total direct costs**	**260,444,808**	**−11,718,813**	**−3,483,803**	**−8,235,010**

*Negative numbers represent savings in favor of first comparator.

*AOM* acute otitis media, *IPD* invasive pneumococcal disease, *PCV-7* 7-valent pneumococcal conjugate vaccine, *PCV-13* 13-valent pneumococcal conjugate vaccine, *PHiD-CV* pneumococcal non-typeable *Haemophilus influenzae* Protein D conjugate vaccine.

Of the three vaccines considered, PHiD-CV was predicted to offer the greatest reduction in direct medical costs, saving US$8.2 million compared to PCV-13. While PHiD-CV was predicted to save US$37,139 less than PCV-13 in direct medical costs for IPD and pneumonia, this was offset by far greater savings in AOM costs. Moreover, according to model projections, the greatest cost burden of pneumococcal disease was treatment of AOM, which accounts for 95% of direct medical costs allocated for the treatment of vaccine-preventable *S. pneumoniae* diseases or US$98.8 million under the PCV-7 strategy. AOM cost savings of the PHiD-CV regimen were predicted to be most influential, introducing an additional saving of US$11.5 million compared to PCV-7 and US$8.2 million compared to PCV-13.

### Incremental cost-effectiveness analyses

Incremental cost-effectiveness analyses were used to compare a PCV-7 vaccination program with those of PHiD-CV and PCV-13. These analyses suggest that a 3+1 schedule of PHiD-CV would dominate equivalent schedules of both PCV-7 and PCV-13. PHiD-CV is expected to dominate over PCV-7 and PCV-13 because of higher potential cost savings and greater QALYs gained.

### Sensitivity analysis

**Figure 3:**
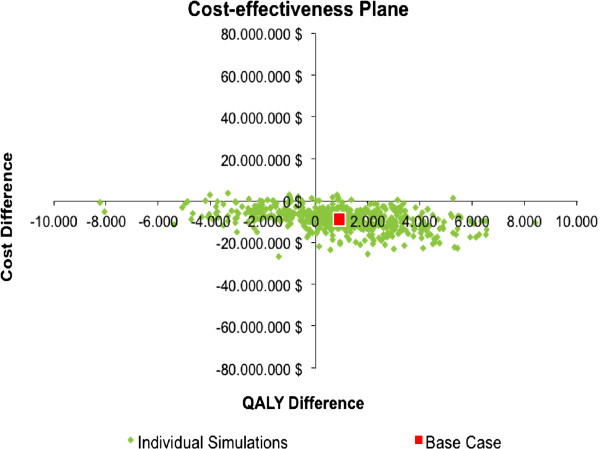
**Probabilistic sensitivity analysis.** Monte Carlo simulation of 1,000 simulations.

The replicates obtained from the probabilistic sensitivity analysis are presented in Figure 3. This analysis indicates that 58.3% of simulations are located in the south-east quadrant, where PHiD-CV dominates PCV-13 in terms of QALYs gained and cost savings, while 2.1% of simulations are located in the north-west quadrant, where PCV-13 dominates PHiD-CV. Implementation of a PHiD-CV strategy results in less costs and provides less QALYs gained compared to PCV-13 in 38.2% of simulations, which are located in the south-west quadrant, and is expected to generate higher more costs and provide greater QALYs gained in only 1.4% of simulations (i.e. north-east quadrant) (Figure 3).

**Figure 4:**
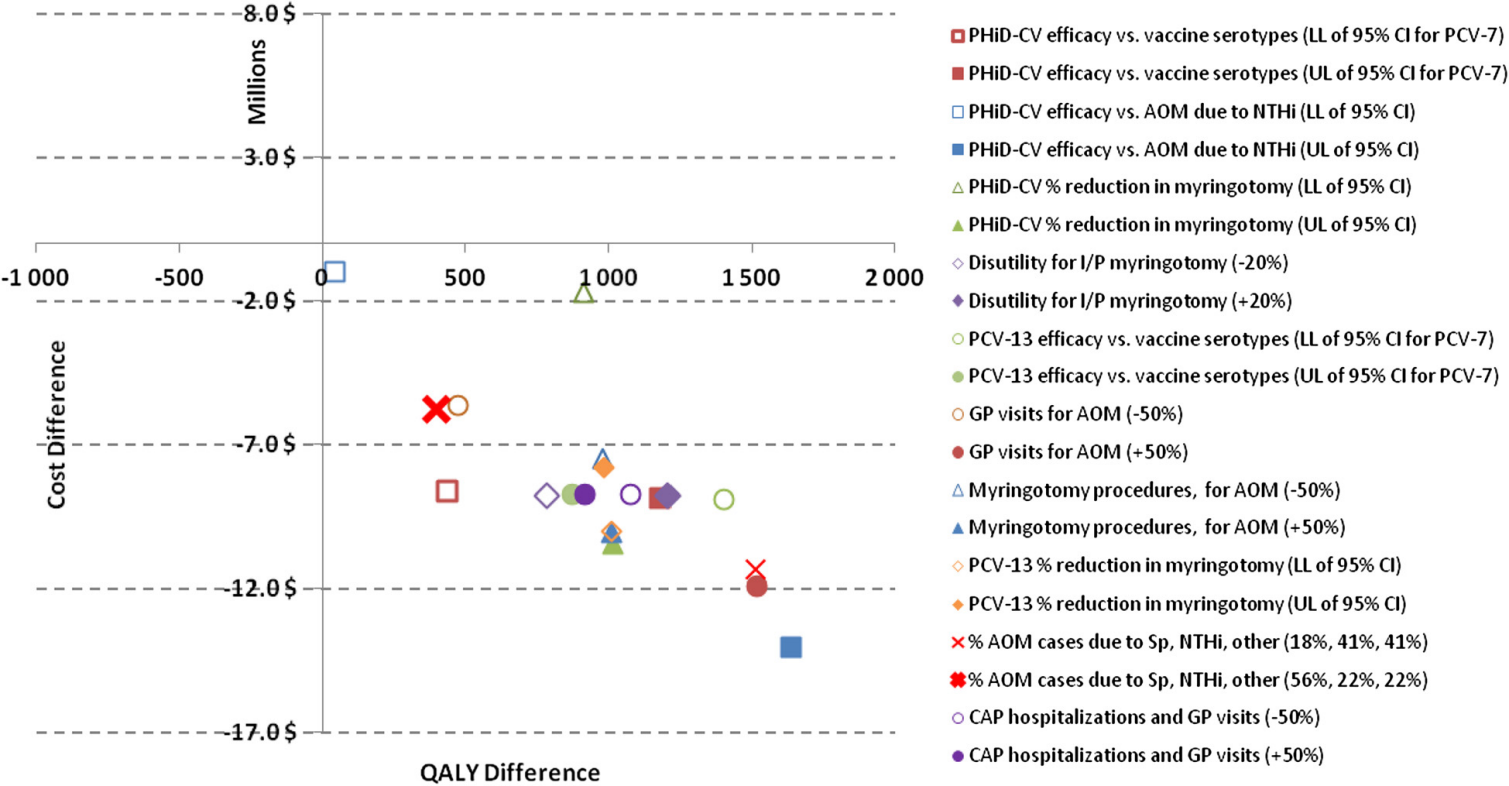
**One-way sensitivity analysis.** AOM = acute otitis media, CAP = community-acquired pneumonia, CI = confidence interval, GP = general practitioner, I/P = inpatient, LL = lower limit, NTHi = non-typeable *Haemophilus influenzae*, PCV-7 = 7-valent pneumococcal conjugate vaccine, PCV-13 = 13-valent pneumococcal conjugate vaccine, PHiD-CV = pneumococcal non-typeable *Haemophilus influenzae* Protein D conjugate vaccine, Sp = *Streptococcus pneumoniae*, UL = upper limit.

While the majority of the model’s variables were found to have very little influence over the conclusion of dominance in Turkey (i.e. that PHiD-CV was less costly and more effective than PCV-13), parameters relating to PHiD-CV efficacy against vaccine serotypes and AOM-related outcomes were found to be the most sensitive in the analysis (Figure 4).

## Discussion

Vaccination, which is the second most effective way (after clean water) to save lives and promote good health [36], saves around 3 million lives each year worldwide [37]; reduces suffering; has vastly reduced the incidence of various diseases; and results in substantial cost savings. In an analysis by the Centers for Disease Control and Prevention in the US, every US$1 spent on immunization saves US$6 in direct medical costs and US$12 in indirect costs [38]. It has also been estimated that each birth cohort vaccinated with the seven main vaccines can save $10 billion in direct medical costs and $33 billion in indirect costs [39].

The benefit of introducing a widespread vaccination program can only be judged on the basis of an accurate estimation of country-specific disease burden. The study reported here adapted a previously described model for the UK [13], but used data from 12 Istanbul hospitals [16] and Turkish Ministry of Health statistics [17] on burden of disease, resource use and costs. These were combined with vaccine efficacy estimates, adjusted to the specific pneumococcal serotype distribution in Turkey [25].

The projections reported herein suggest that introducing either PCV-13 or PHiD-CV would greatly reduce pneumococcal infection disease burden compared with PCV-7. Not surprisingly, both the PCV-13 and PHiD-CV vaccines were also predicted to have a greater beneficial impact on cost savings due to pneumococcal disease and AOM. These observations are consistent with those made by other authors [13,40,41].

The modeled projections indicate that PCV-13 is expected to offer a greater impact on IPD and pneumonia (by virtue of its greater complement of *S. pneumoniae* serotypes) than PHiD-CV. This translates into a saving of one additional death every year after the launch of a vaccination program (65 LYs gained over a 1-year period at a vaccine steady state). However, in terms of cost and QALYs, this relative reduction in IPD and pneumonia was grossly outweighed by the substantial reduction in AOM cases estimated with PHiD-CV. Moreover, when comparing the cost profiles of the two vaccines, the additional costs saved by PCV-13 in IPD and pneumonia (around US$37,139) were approximately 200-fold less than the additional savings in AOM estimated with PHiD-CV (around US$8.2 million). The relatively greater impact of PHiD-CV on AOM also contributed to the prediction that the vaccine would save 933 more QALYs than PCV-13 over the 1-year period. It was these substantial differences in direct cost savings and quality of life that led to PHiD-CV being identified as the most cost-effective vaccine under the modeled conditions.

The observation that AOM treatment costs drive the cost-effectiveness of PCVs in children <10 years of age is as expected, given the substantial prevalence of the disease within this age group. Previous estimates suggest that >80% of children will experience ≥1 episode of AOM by the age of 3 years [42]. AOM is a major burden for healthcare systems, resulting in around 1.7 times as many hospitalizations as pneumonia in children aged 0–9 years in the UK; and 73 times as many GP consultations [43]. Further reducing the incidence of AOM compared with PCV-7 was estimated in this study to decrease the number of AOM hospitalizations by 7,956 and 2,234 for PHiD-CV and PCV-13, respectively, and the number of GP visits by 267,212 and 75,019, respectively. AOM burden of disease is reflected by the sizeable annual cost of AOM treatment predicted under all three of the vaccination schedules considered in the model, ranging from US$87 million to US$99 million. One limitation of this study is that AOM incidence was not available in Turkey, therefore data from other countries were used to estimate the prevalence of AOM in Turkey [8,18,19]. A separate scenario was analyzed to test the base case incidence estimate of 33,000 per 100,000 person years obtained from Garcés-Sánchez et al. study [18]. The base case incidence rate of AOM was reduced by - 50% in 0–5 and 5–9 age group down to 16,500 and 10,095 per 100,000 person respectively. In this scenario PHiD-CV was projected to dominate PCV-13 as it generates 491 more QALY and US$5.5 million cost savings.

Historically, researchers have been intrigued by the differences in efficacy of pneumococcal vaccines against clinical AOM between the POET [7] and FinOM [35] trials. However, a direct comparison between the 34% overall impact against AOM in the POET trial (11-valent pneumococcal polysaccharide conjugate vaccine conjugated to *H. influenzae-*derived protein D [11-Pn-PD]) versus 7% in FinOM (PCV-7) cannot be made due to the different settings and designs of these trials. Palmu et al. [44], former investigators of FinOM, assessed the impact of variations in case definition, design and local epidemiology between trials, and concluded that these factors only account for part of the difference. An extension of this re-analysis with the POET data set, using a standardized case definition, confirmed that the original difference in case definition was not the cause of the observed AOM efficacy difference. De Wals et al. [45] assessed various scenarios, adjusting for different factors. Replacement due to non-vaccine serotypes, not observed in POET, was identified as a main factor to explain the divergence. The observed reduction in AOM episodes due to NTHi in POET with the 11-Pn-PD vaccine was identified as a secondary factor to explain the divergence. Tangible differences in calculated maximal efficacy against AOM are thus consistent with those presented in independent evaluations [46].

The vast amount of antibiotics prescribed for childhood AOM has the potential to increase antibiotic resistance [47], which may increase treatment costs and decrease quality of life. The prevention of AOM by vaccination therefore has an important role in the reduction of antibiotic prescriptions [42]. However, while reducing the number of AOM cases is likely to reduce the rate of antibiotic prescription, modeling the impact of vaccination in this context is complex and was beyond the scope of the analysis reported here.

The predictions of all simulation models are dependent upon the approximations and assumptions used to configure them. Where possible, we endeavored to use region-specific surveillance data and information from controlled clinical trials. However, assumptions were required for certain parameters in the model. In the absence of any specific efficacy data, the efficacy of the additional serotypes in PHiD-CV and PCV-13 had to be estimated using the average efficacy of the serotypes in PCV-7, but their true efficacy may lie at either extreme of the efficacy range (87% for 19F to 99.9% for 9V). However, this assumption is in line with WHO guidelines and does not appear to overtly bias the model [13,48].

A second approximation in the model is the absence of indirect vaccine effects (e.g. herd protection and serotype replacement), which is an important limitation. PCV-7 has been shown to reduce nasopharyngeal carriage of vaccine-type *S. pneumoniae* serotypes in vaccinated and unvaccinated individuals [49-51]. PCVs are therefore capable of having an additional impact on the overall transmission of *S. pneumoniae* within the population, which would potentially translate into protection for the unvaccinated population [52-54]. Lessons from the large-scale PCV-7 vaccination program in the US indicate that vaccination can result in substantial indirect herd effects, e.g. IPD has been reported to have decreased by 15% in unvaccinated children aged <5 years [14], and by 29% in unvaccinated children >5 years and adults [55]. Most deaths from pneumococcal disease occur in elderly adults [56], as they are more likely to have compromised immune systems. Excluding a herd effect on the elderly is therefore likely to have resulted in an underestimation of the true health gains that a PHiD-CV, PCV-7 or PCV-13 vaccination program could achieve. Indeed, indirect effects that increase the number of IPD cases averted may have a crucial impact on economic savings per QALY gained [19,57,58]. Opposing this effect is serotype replacement, which results in increased disease from non-vaccine serotypes [59]. At present in the US, beneficial herd effects appear to outweigh negative serotype replacement [14,59]. Indirect effects of the vaccines (herd effect and serotypes replacement) were not included. Since PCV-7, PHiD-CV and PCV-13 are directly compared and the potential differences in indirect effect induced by each vaccine are not known, the inclusion or exclusion of equal (not differential) indirect effect for all vaccines won’t impact the results, because the incremental differences between three vaccines would be the same with inclusion or without inclusion of indirect effect. On the other hand herd protection plays an important role in impact on cost-effectiveness ratio when the vaccines are compared to a no vaccination strategy.

Another weakness of the current analysis is that long-term complications after meningitis (neurological sequelae and severe hearing loss) were not included, due to the absence of Turkey-specific incidence and cost data. However, if available incidence data are used (neurological sequela 7% [60]; hearing loss 13% [61]), this would result in six fewer cases of long-term complications for PHiD-CV vs PCV-7 and eight fewer for PCV-13 vs PCV-7. This small number of cases is not expected to influence the outcome or conclusions of this study.

Additional assumption is related to the extrapolated incidence rates of IPD, CAP and AOM in 5–9 age group. This assumption may have a minor impact on the results and conclusions, since in 0–10 age group approximately 90% of IPD, CAP and AOM cases are occurring in children below 5 years of age.

Another important approximation is related to the modeling of the impact of the vaccines based on the IPD serotypes distribution before PCV-7 implementation. The pre PCV-7 IPD serotypes distribution was used in a base case in order to have a consistent data set of both incidence rates and serotypes distribution from the same time period of pre PCV-7 introduction before 2008. The impact of PCV-7 is projected using the model. An alternative scenario was analyzed using the latest serotypes distribution from Ceyhan et al. and estimating the impact of vaccination on costs and QALYs gained [62]. In the scenario analysis PHiD-CV was projected to yield 759 more QALYs and cost savings of US$11.6 million when compared with PCV-13. The analysis described here assumes a 3+1 dosing schedule in order to gain maximum impact from the vaccination program. Although some countries have adopted 2+1 schedules of PCVs [63], the 3+1 schedule is used in Turkey. Our decision to focus on 3+1 schedules of PCVs was also based on a previous cost simulation using UK statistics, which showed that implementing a 3+1 schedule had incremental health benefits over the 2+1 schedule for PCV-7 currently in place in the UK [45]. This four-dose program is in line with the original recommendations for vaccination with PCV-7 and is supported by data demonstrating that efficacy increases with increasing numbers of doses [26].

With over 70 million people, Turkey has the 17th largest population in the world and the 3rd largest in Europe [64]. Turkey’s huge population is diverse and heterogeneous, with discrepancies in income, poverty, infrastructure, and services between those living in the east and the west, and between those in urban and rural areas [65]. This analysis has shown that the use of PHiD-CV and PCV-13 are more cost-effective than PCV-7 (in terms of more QALYs gained and costs saved) in this upper middle income country. These results are in line with other cost-effectiveness studies of PCVs of various valencies in high (Canada [46]) and low (The Gambia [66]) income countries, suggesting that these results may be applicable to the other, economically heterogeneous, countries in the Middle East and North Africa (MENA) region, assuming similar disease incidence rates and treatment costs.

## Conclusions

The health economic analysis described here estimates the relative cost-effectiveness of PHiD-CV, PCV-13 and PCV-7 vaccination programs in Turkey, using currently available vaccine efficacy data and region-specific information on the burden of pneumococcal disease. Projections of direct medical costs show that a vaccination program with any of these vaccines would have a significant impact on the economic burden of invasive and non-invasive pneumococcal disease amongst children aged <10 years. Over a 1-year period at vaccine steady state, PHiD-CV and PCV-13, according to model outputs, have approximately equivalent impact on mortality associated with IPD and pneumonia, while PHiD-CV is projected to prevent approximately three times as many GP visits for AOM compared to PCV-13. Overall, PHiD-CV is projected to dominate PCV-13, given its potential to generate more QALYs and greater estimated healthcare cost savings.

## Competing interests

Drs Bakır and Türel declare that they have no competing interests. Mr Topachevskyi is an employee of GlaxoSmithKline group of companies. GlaxoSmithKLine Biologicals SA financed this study and article, including the article-processing charge.

## Authors’ contributions

MB was involved in data collection and analysis and reviewed the manuscript. OTü participated in data collection and review. OTo contributed to the modeling, data analysis, editing and drafting the manuscript. MB had full access to the data and had final responsibility for submission for publication. All authors read and approved the final manuscript.

## Disclosure

Parts of these data were presented as a poster at the World Society for Pediatric Infectious Diseases - 6th World Congress, November 18–22, 2009, Buenos Aires, Argentina.

## Pre-publication history

The pre-publication history for this paper can be accessed here:

http://www.biomedcentral.com/1472-6963/12/386/prepub
